# A Novel Untethered Hand Wearable with Fine-Grained Cutaneous Haptic Feedback

**DOI:** 10.3390/s22051924

**Published:** 2022-03-01

**Authors:** Alexander Co Abad, David Reid, Anuradha Ranasinghe

**Affiliations:** 1 Faculty of Science, School of Mathematics, Computer Science, and Engineering, Liverpool Hope University, Liverpool L16 9JD, UK; reidd@hope.ac.uk (D.R.); dissana@hope.ac.uk (A.R.); 2 Department of Electronics and Computer Engineering, De La Salle University, 2401 Taft Avenue, Manila 0922, Philippines

**Keywords:** haptic devices, cutaneous feedback, medical training, virtual reality, wearable devices, teleoperation

## Abstract

During open surgery, a surgeon relies not only on the detailed view of the organ being operated upon and on being able to feel the fine details of this organ but also heavily relies on the combination of these two senses. In laparoscopic surgery, haptic feedback provides surgeons information on interaction forces between instrument and tissue. There have been many studies to mimic the haptic feedback in laparoscopic-related telerobotics studies to date. However, cutaneous feedback is mostly restricted or limited in haptic feedback-based minimally invasive studies. We argue that fine-grained information is needed in laparoscopic surgeries to study the details of the instrument’s end and can convey via cutaneous feedback. We propose an exoskeleton haptic hand wearable which consists of five 4 × 4 miniaturized fingertip actuators, 80 in total, to convey cutaneous feedback. The wearable is described as modular, lightweight, Bluetooth, and WiFi-enabled, and has a maximum power consumption of 830 mW. Software is developed to demonstrate rapid tactile actuation of edges; this allows the user to feel the contours in cutaneous feedback. Moreover, to demonstrate the idea as an object displayed on a flat monitor, initial tests were carried out in 2D. In the second phase, the wearable exoskeleton glove is then further developed to feel 3D virtual objects by using a virtual reality (VR) headset demonstrated by a VR environment. Two-dimensional and 3D objects were tested by our novel untethered haptic hand wearable. Our results show that untethered humans understand actuation in cutaneous feedback just in a single tapping with 92.22% accuracy. Our wearable has an average latency of 46.5 ms, which is much less than the 600 ms tolerable delay acceptable by a surgeon in teleoperation. Therefore, we suggest our untethered hand wearable to enhance multimodal perception in minimally invasive surgeries to naturally feel the immediate environments of the instruments.

## 1. Introduction

There has been a rapid increase in the multidisciplinary study of haptics in the last two decades among psychophysics, experimental psychologists, and engineers in the field of mechanical design, electronics, automation, and computer science [1]. In 2017, a comprehensive review on haptics and its applications to different disciplines are reported by Sreelakshmi and Subash. They listed five common haptic devices, namely, Haptic Paddles, Haptic knobs, Novint Falcon, Force Feedback Gaming Joysticks, and SensAble’s Omni Phantom, and they also listed haptic technology applications such as vision substitution for the visually impaired, haptics in the automotive industry, virtual education, research, medicine, arts and design, holographic interaction, biometrics, and e-commerce [2]. A more recent application-based review of haptics technology was published by Giri et al. in 2021, in which they classified the application of haptic devices based on construction, functionality in various fields, prospects, and major limitations related to haptics technology [3]. Giri et al. presented a comprehensive list of commercially available haptic devices used in VR applications, telerobotics, and telemedicine.

The study of haptic feedback can be divided into two: kinesthetic and cutaneous. Kinesthetic feedback can also be described as proprioception, and it refers to awareness or sense of touch created from muscle tensions with the help of sensory receptors [3]. Kinesthetic feedback is about the position and velocity of neighboring body parts as well as the applied forces in the muscles and joints [4]. On the other hand, cutaneous feedback, also known as tactile feedback, refers to the information obtained from various mechanoreceptors on the skin [3] enabling humans to recognize an object’s properties such as shape, edges, and texture [5].

Haptic devices can be input devices (haptic sensors) or output devices (haptic actuators). Research and development on haptic devices to facilitate interaction with VR objects or remote environments and to enhance the immersive experience have been taking place at a rapid rate [6,7,8,9]. Naturalistic interaction with real or VR environment is a significant factor in the success of spatial computing that aids cooperation, communication, and integration between humans and robots [6,9]. From the point of view of users, comfort and wearability are significant factors in designing haptic devices. Wearability can be defined as the combination of weight, shape, form-factor, functionality, and ergonomics [6,7]. Haptic feedback for wearables can be kinesthetic, cutaneous, or the combination of both cutaneous and kinesthetic feedback [1,6,7].

**Figure 1:**
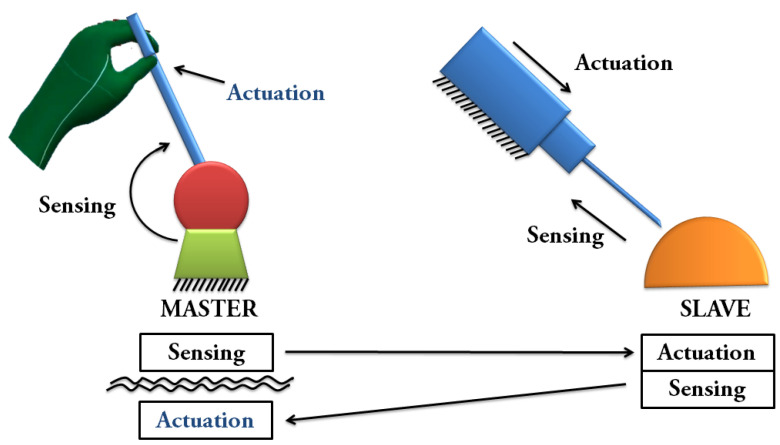
Teleoperation system with ungrounded cutaneous feedback to the operator based on the diagram by Paccheirotti et al. [5]. The cutaneous feedback to the human operator gives information about the forces exerted at the slave side and does not affect the stability of the control loop [5].

Most of the popular haptic devices used in telerobotics and telemedicine, such as Omega and Phantom, provide kinesthetic feedback for single point contact interaction [4]. Moreover, according to Mehrdad et al., one of the main challenges in haptic-enabled telerobotic systems is the communication delay which causes instability and divergence. Our proposed high-cutaneous haptic hand wearable has an average latency of 46.5 ms, which is much lower than the 600 ms tolerable delay acceptable by a surgeon in the absence of haptic feedback reported by Tavakoli and Patel [10]. Stability is one of the main bottlenecks of adding haptic feedback to surgical telerobotic systems [11]. However, by delivering ungrounded cutaneous feedback to the human operator, Pacchierotti et al. demonstrated that cutaneous haptic feedback could be used to enhance the performance of robotic teleoperation systems while guaranteeing their safety, even in the presence of destabilizing factors such as communication delays and hard contacts [5], as shown in the diagram in Figure 1.

Cutaneous feedback can be in the form of vibration produced by mini-vibration motors [12,13,14,15,16], skin stretch or tangential motion to the skin [17], and tactile displays that produce tapping motion creating vertical movements towards the skin forming a matrix of tactile pixels (taxel) [18] that triggers tactile mechanoreceptors: Meissner’s corpuscles and Merkel’s cells that are sensitive to edge pressure and flutter tap as reported by Visell [19]. Tactile matrix displays are often used as vision substitution but can also be used as a tactile augmentation to a vision system similar to in VR and telerobotics applications. Tactile displays can be made from different forms of tactile actuators (tactors) such as solenoids [18,20,21,22,23,24], piezoelectric [25,26,27,28], voice coil motors [29], shape memory polymer (SMP) [30], smart memory alloy (SMA) [31,32], stepper motor [33], and pneumatic [34,35]. The performance and effectiveness of teleoperation and immersive systems can be enhanced by cutaneous feedback that provides an effective way to simplify the design of a haptic wearable [6]. One of the goals of haptics research is to develop an effective and efficient tactile “display” for a human–machine interface that can reproduce as closely as possible the natural feel of an object. Tactile displays are not only for vision substitution systems [20,25], but they can also be used to enhance the immersive experience in telecommunications or teleoperations [36,37], biomedical engineering [6], telerobotics [38], material recognition [39], online shopping [7], human–computer interaction (HCI) [19,40,41], and VR environments [18,24].

In 2007, King et al. [42] demonstrated a tactile matrix display in the form of a 3 × 2 pneumatic balloon actuator array that can be used as cutaneous haptic feedback for surgical robotic tools. This balloon actuator display is suitable for mounting on the master controls of the da Vinci Surgical System [43]. Although the fingerpad is compact, flexible, and lightweight, the overall setup is bulky, heavy, and not easily portable because it includes electropneumatic pressure regulators and air supply. Aside from the balloon actuator array, there are other cutaneous haptic feedbacks applied to surgical robotic tools, such as the vibrotactile by McMahan et al. (2011) that can be added to Intuitive Surgical’s existing da Vinci Surgical System to provide vibrotactile feedback of tool contact accelerations [44], the skin stretch or tangential motion cutaneous haptic feedback for grasp control in laparoscopy demonstrated by van der Putten et al. [17], and the fingertip skin deformation cutaneous haptic feedback in robot-assisted surgery presented by Meli et al. [45] in 2014. Pacchierotti et al. presented in 2016 a cutaneous feedback of fingertip that combined fingertip skin deformation and vibrotactile feedback for palpation in robotic surgery while guaranteeing the teleoperator’s stability [46]. They implemented their cutaneous feedback solution on an Intuitive Surgical da Vinci Standard robot.

In the da Vinci robot, force feedback is given to the joystick or master controller whenever the surgical instrument or slave device is in contact with the medical phantom or actual body organ. However, fine-grained wearable haptic cutaneous feedback from the medical phantom in the form of vibration or texture can help the surgeon control the device in a more stable manner as compared to force feedback only [46]. Force feedback is limited to conveying information about the medical phantom in da Vinci setup. However, we would introduce fine-grained cutaneous feedback from the medical phantom to the surgeon using our proposed untethered hand wearable cutaneous haptic device.

Spatial computing research explores the wearability and portability of haptic devices [6,7,21,47] that give cutaneous feedback. This can directly affect the immersive experience [18,21,23]. The realization of a compact fingertip tactile display aiming at integration with a kinesthetic or force feedback system is a real challenge [7,48]. Much research is still needed to reduce the form-factor of tactile displays without compromising transducer strength, spatial resolution, and bandwidth [21]. The review paper from Pacchierotte et al. [6] on wearable haptic systems for the fingertip and hand in 2017 presents a taxonomy of wearable fingertip systems comparing their actuation technology, weight, and dimensions at the fingertip.

**Figure 2:**
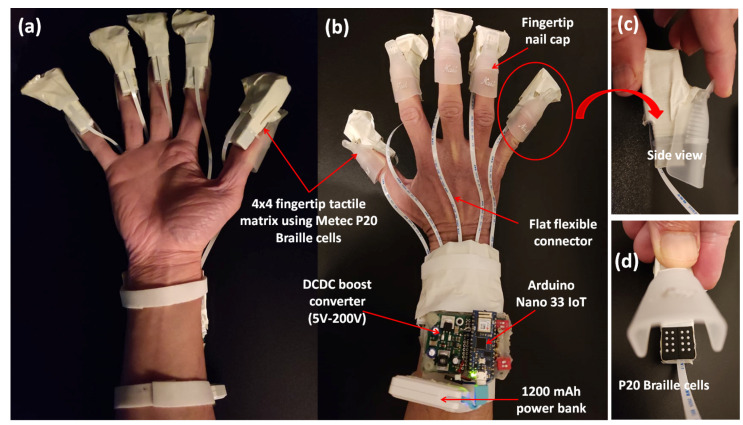
Untethered high-resolution haptic hand wearable having 80 tactile actuators (tactors). There is a 4 × 4 matrix of tactors on each fingertip made from Metec P20 Braille cells. The prototype has an open-backhand and open-palm design for easy hand tracking. (**a**) Open-palm view, (**b**) open-backhand view, (**c**) side view of the fingertip tactile matrix with an invisible rubber band to increase the grip of the nail clip if the user has a small or thin finger, and (**d**) 4 × 4 fingertip tactile matrix made from two P20 Braille cells.

In this study, we propose a unique solution, which is an untethered high-resolution cutaneous haptic hand wearable as shown in Figure 2. Our prototype is a compact, lightweight (204 g), battery-powered (maximum power consumption of 830 mW), wireless (Bluetooth and WiFi-enabled with an average latency of 46.58 ms), modular, and high-resolution cutaneous feedback device having 80 tactile actuators distributed on the fingertips. The 80 piezo-based pin actuators are made from Metec P20 Braille cells [49]. The whole system’s total weight, including the small USB power bank, is 204 g. The controller board at the arm is 72 g, and each fingertip module is just 19 g. The fingertip module of our prototype is lighter than any of the fingertip tactile displays with pin array or pin matrix listed by Pacchierotte et al. in 2017 [6].

Our untethered high-resolution haptic hand wearable with detailed parts is shown in Figure 2b. Each fingertip module has a nail clip for easy mounting on the fingertip and a small clear rubber band, as shown in Figure 2c, to increase the grip if the user has a small or thin finger. In our prototype, the use of a nail clip and a small rubber to fit our fingertip module securely to any size and shape of a fingertip is much simpler compared to the method of personalizing a wearable for target fingertip by taking into account its specific geometrical characteristics as well as some target performance metrics made by Malvezzi et al. [50]. The exposed 4 × 4 matrix made from two P20 Braille cells is shown in Figure 2d. The components or modules in our prototype are connected or combined using sockets and detachable cables. We used thin and light-weight flat flexible cables (FFC) in connecting our fingertip modules to the control board. Depending on the application, we can reduce the number of fingertip modules. For example, a pinching application needs only two fingertips (index and thumb), while grasping needs five fingers. This modular property adds to the novelty and flexibility of our prototype for easy mount or replacement of parts. The connecting cables are flexible enough to allow the fingers to flex, allowing a grasping hand gesture.

Our prototype operates at a 5 V DC supply and consumes a maximum current of 166 mA when all the fingertip matrices are activated and connected via Bluetooth. Each fingertip module consumes around 3 mA of current. Using 1200 mAh rechargeable battery, our prototype can be used for six hours. The maximum power consumption of our device is just 830 mW. Our untethered prototype has an open-backhand, and open-palm thimble design similar to weart TouchDIVER [51] for easy hand tracking, but unlike the TouchDIVER that has only three fingertip modules, our prototype has five fingertip modules. It has an open-palm design similar to the Dexmo Haptic Force-feedback Gloves [52], and BeBop Forte Data Gloves [53]. Our prototype can also enhance and complement the above force-feedback haptic and data gloves by providing high-resolution cutaneous feedback. The BeBop data glove offers six vibrotactile feedbacks, while our prototype has 80 tactile feedback actuators. Moreover, our prototype can produce not only “up” or “down” tactile feedback from the P20 Braille cells but also different tapping or vibration patterns at different frequencies with varying duty cycles. Unlike Dexmo and BeBop gloves, our prototype is made from commercial off-the-shelf (COTS) low-cost components. Though our 80-tactile actuators are lower than the HaptX Gloves DK2 haptic VR gloves that have 133 tactile feedback points per hand [54], we have the advantages of untethered, compact, battery-powered, modular, and lightweight design. To the best of our knowledge, our prototype has the novelty of having the highest number of tactile feedback actuators in an untethered, portable, open-palm haptic glove design that can fit any size of hand because of its flexibility and adjustability.

**Figure 3:**
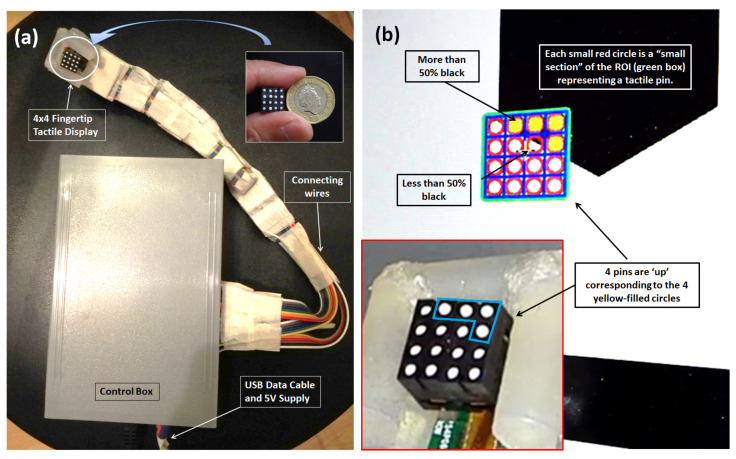
Our initial miniaturized fingertip module presented in 2020, 4 × 4 fingertip tactile matrix actuator with edge detection scanning ROI simulator presented in [55]. (**a**) Hardware setup, (**b**) graphical user interface (GUI).

In our initial studies in 2020, we demonstrated a 4 × 4 fingertip tactile matrix display that can be strapped on a finger [48], as shown in Figure 3. It is made from commercially available dot Braille cells [55]. Our previous prototype has a 1.08 cm${}^{2}$ surface area, a pin pitch of 2.6 mm, and uses a 5 V DC supply, but it is bulky, with its control box containing the microcontroller circuitry, as shown in Figure 3a. Each tactile pin is a small solenoid that controlled by a microcontroller with an h-bridge motor driver. The tactile matrix actuator is connected to a tactile matrix simulator, as shown in Figure 3b, that scans binary images and uses a Canny edge algorithm for edge detection. Sixteen regions of interest (ROIs) form a 4 × 4 matrix in the simulator that corresponds to the 16 tactile actuators. The integration of the hardware prototype and the tactile matrix simulator allows the user to feel the surface or the edges of binary image as the scanning ROI moves across the computer display.

In this study, we managed to expand it into a five-fingertip, wireless, high-resolution cutaneous haptic wearable. We used a different commercially available Braille cell that uses piezo-based actuators. We used the Metec P20 Braille cell [49] that has a compact and lightweight backplane containing shift register chips that can interface easily with a microcontroller. Our prototype has a fine-grained 4 × 4 matrix of cutaneous stimuli on each fingertip. The hardware section of this paper provides more details on the specifications of this Braille cell.

In our current study, 80 tactile actuators were brought to hand-mounted size in Figure 2b from a bulky controller in Figure 3a by introducing Arduino Nano 33 IoT. The 80 tactile actuators can be controlled individually using a small microcontroller (Arduino Nano 33 IoT) in real time. We used Processing software to create a simple application to demonstrate the hardware capability of our prototype. Processing is an open-source software with an OpenCV library that we used in image processing and edge detection. We developed a simulator for five 4 × 4 tactile matrices corresponding to the tactile actuators for the fingertips. The tactile matrices in our simulator can be moved using a computer mouse using the coordinates of its pointer as a reference. The tactile matrices can also be anchored on the fingertips and wrist coordinates extracted in real time by the hand-tracking algorithm of the Leap Motion Controller. Moreover, we created a 3D VR environment using Unity software. Using an Oculus Quest 2 VR headset that has builtin hand-tracking capability, we used our prototype to feel the surface or edges of a 3D VR object. The tabulated comparison between our previous wearable and our current prototype is shown in Table 1.

This paper is structured as follows: overall schematic diagram is shown in Section 2, construction of the prototype is shown in Section 3, experimental setup is detailed in Section 4, application example is given in Section 5, experiments involving human participants are detailed in Section 6, limitations of the current design and possible future improvements are discussed in Section 7, and, lastly, conclusion and recommendation are given in Section 8.

## 2. Overall Schematic Diagram

**Figure 4:**
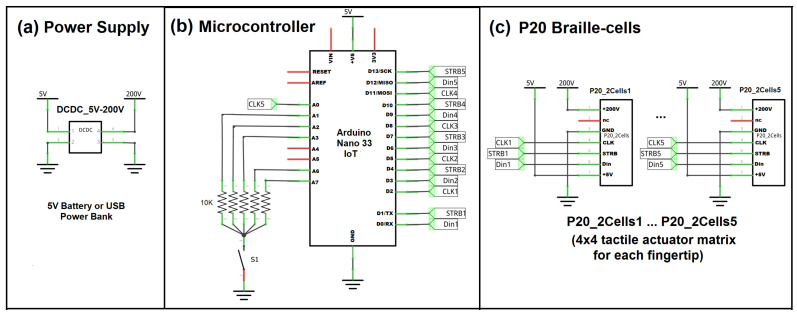
Whole system schematic of the untethered high-resolution haptic hand wearable: (**a**) power supply block, (**b**) microcontroller block, and (**c**) P20 Braille cells block.

The whole system schematic diagram is shown in Figure 4. This is the bird’s eye view or the blueprint of the prototype. The whole system can be divided into three blocks, namely, power supply, microcontroller, and P20 Braille cells.

The power block as shown in Figure 4a is composed of a battery or USB power bank, and a DC–DC boost converter 5–200 V DC supply for the P20 Braille cells piezo-based actuators. The main controller of the system is the Arduino Nano 33 IoT [56], and its schematic is shown in Figure 4b. It has 14 digital pins (D0–D13) and 8 analog pins (A0–A7). The analog pins can be used as digital output pins when needed. Among the seven analog pins of Arduino Nano 33 IoT, analog pins A4 and A5 have internal pull-up resistors for I2C communications. These A4 and A5 analog pins are floating or free, as shown in the schematic in Figure 4b, and they can be used in the future for I2C modules such as DRV2605L Haptic Motor Controller [57] for driving different types of mini vibration motors. In this study, the analog pin A0 was declared as a digital output pin and connected to one of the P20 Braille cells.

We used switchable pull-down resistors for analog pins (A1, A2, A3, A6 and A7) as shown in Figure 4b as a provision for future tests if there is a need to read analog signals. The switch can be turned on if the five analog pins with pull-down resistors are connected to variable resistors such as flex sensors, or can be turned off, as in this study, if they are to be used as digital output pins. This switch adds flexibility in the usage of analog pins. It is also worth mentioning that the Arduino Nano 33 IoT has a virtual serial port wherein the USB connector of the board connects directly to the USB host pins of the SAMD21 [58]. The D0 and D1 digital pins are considered Serial1 but are declared digital output pins in this study.

Each of the P20 Braille cells has a shift register in its active backplane, and the schematic is shown in Figure 4c. The active backplane has supply pins for 200 V, 5 V and GND, and three digital control pins, namely, data-in (Din), strobe (STRB), and clock (CLK). The digital pins are connected to the microcontroller. Fifteen digital output pins are needed to control the five sets of 4 × 4 fingertip tactile matrix. Analog pin A0 declared as digital out pin completes the 15 control pins for fingertip tactile actuator matrices.

## 3. Construction of the Prototype

### 3.1. Hardware

The palm view and backhand view of the high-resolution haptic hand wearable prototype are shown in Figure 2a,b, respectively. The Arduino Nano 33 IoT microcontroller and DC–DC boost converter module are shown in Figure 2b. The haptic hand wearable follows an open-palm design inspired by BeBop Forte Data Gloves [53]. The fingertip tactile matrices are mounted on each finger using a nail clip. The whole prototype is made from different commercial-off-the-shelf electronic breakout boards or modules integrated using sockets and plugs for easy mount or replacement parts. More details of each component are discussed in the following sections.

#### 3.1.1. Microcontroller

**Figure 5:**
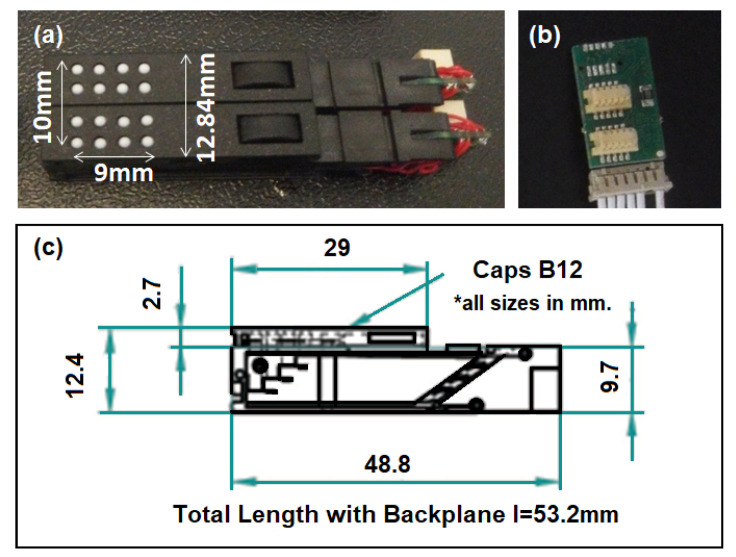
P20 Braille cell. (**a**) The 4 × 4 matrix from two pieces of P20 Braille cells, (**b**) two-position backplane for two P20 Braille cells, and (**c**) P20 side-view dimensions [49].

The prototype uses Arduino Nano 33 IoT microcontroller, shown in Figure 5b, that is mounted on a socket to be removed or replaced easily. This small microcontroller has a built-in Bluetooth, BLE, and WiFi module for wireless connectivity. It also has a built-in six-axis inertial measurement unit (IMU). The pins of this microcontroller operate at 3.3 V and are not 5 V-tolerant.

According to a global Arduino forum moderator [59], the u-blox NINA-W102 WiFi module on the Nano 33 IoT uses an ESP32 microcontroller. The Nano 33 IoT has an ATSAMD21G18 and an ESP32 combined. The code runs on the ATSAMD21G18, which communicates with the ESP32 running a firmware Arduino wrote [60]. The ESP32 inside the Arduino Nano 33 IoT can be used for WiFi, BLE, and classic Bluetooth. Because of this capability, an external Bluetooth module and WiFi module are not necessary for this study.

#### 3.1.2. DC–DC Converter

The piezo-based tactile actuators of P20 Braille cells operate at a 200 V supply. A DC–DC boost converter is needed to raise the 5 V DC supply to 200 V [61] that can be purchased from Metec together with the P20 Braille cells. It has a printed circuit board (PCB) with dimensions of 26 mm × 38 mm.

#### 3.1.3. Tactile Actuator

There are two general types of tactile actuators used in the prototype: piezo-based and electromagnetic-based actuators.

##### P20 Braille Cell

The P20 Braille cell from Metec has eight dots driven by piezo-actuators (bending type) [49]. It has the smallest form factor among the Braille cells of Metec. They can be bought in a complete package together with an active backplane with connecting cable and the DC–DC 5–200 V power supply. A 4 × 4 tactile matrix with 9 mm × 10 mm tactile surface can be made by combining two P20 Braille cells, as shown in Figure 5a. According to the datasheet of the P20 Braille cell, the cells can be stacked to create a larger Braille display. However, this study needs only to combine two P20 Braille cells for each fingertip using a two-position backplane as shown in Figure 5b. The side-view dimensions of the P20 Braille cell [49] are shown in Figure 5c. The weight of a single cell is just 4.19 g.

The total weight of the 4 × 4 fingertip tactile matrix, including the nail clip, is 19 g, which is lighter than most of the fingertip actuators presented in [6]. Each of the piezo-based tactile pins can be controlled in a static “up”, and “down” position or can be activated in a tapping mode wherein frequency, duty cycle, and duration of the vibration can be varied.

#### 3.1.4. Shift Register

The active backplane for the P20 Braille cells has a 16-bit high voltage capable shift register integrated chip HV509. One chip is enough to control two P20 Braille cells. Though Metec granted our request to cut the eight-position active backplane into two-position, it was difficult to expose the data out pin due to the small surface mount devices in the circuitry. It should have been easy to cascade or control the five 4 × 4 matrices using only three digital Arduino pins. Because of this, each of the 4 × 4 fingertip tactile matrices has been treated as individual 16-bit registers.

#### 3.1.5. Leap Motion Controller for Hand Tracking

The Leap Motion Controller is used as a hand tracker in this study. This is a small, fast, and accurate optical hand tracking device that captures the movement of hands and fingers. It has a dimension of 80 mm × 30 mm × 11.30 mm and weighs 32 g. The controller can track hands within a 3D interactive zone that extends up to 60 cm ($24^{\prime \prime }$) with a 140 × 120° typical field of view. Leap Motion’s software can identify 27 hand elements, including bones and joints. It can track them even when they are obscured by other parts of the hand [62]. The fingertip and wrist coordinates from the Leap Motion Controller are the reference points of the 4 × 4 tactile matrices of our simulator.

### 3.2. Software

**Figure 6:**
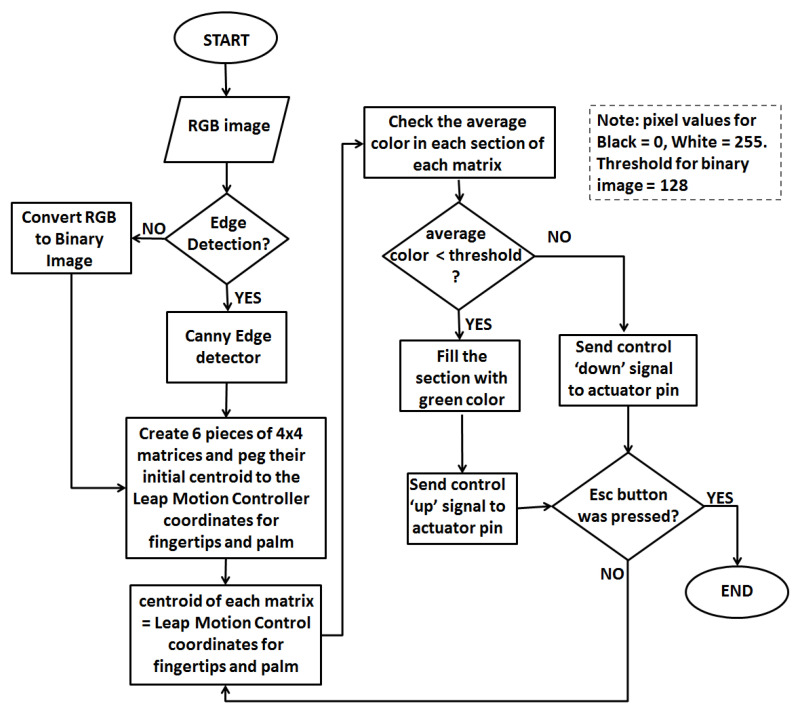
ROI algorithm flow chart.

The software in this study is divided into two categories: firmware and application. The firmware is the software used to program the microcontroller, while the application software is about the graphical user interface (GUI) or the tactile matrix simulator. The following sections will provide more discussions on these two categories. The flow chart for the software algorithm is shown in Figure 6.

#### 3.2.1. Firmware

The firmware is written in C/C++ and compiled using the Arduino IDE. The firmware deals with data framing and an algorithm that controls the signals to activate the tactile actuators. It controls the operation of the 8-bit shift registers connected to the tactile actuators. There are two 8-bit shift registers for each 4 × 4 tactile matrix. Each cell is connected to an 8-bit shift register and can be controlled using one-byte data or a three-digit cell variable that can hold 000 to 255.

#### 3.2.2. Tactile Matrices Simulator

**Figure 7:**
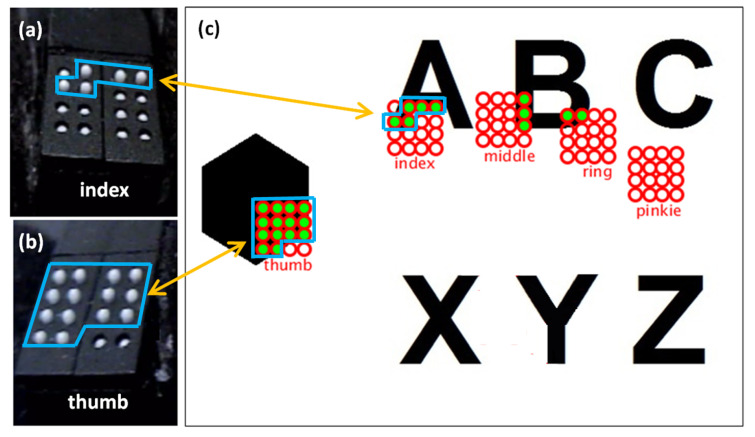
Fingertip tactile matrices, and graphical user interface (GUI) developed using Processing. The activated pins of (**a**) index fingertip and (**b**) thumb tactile matrices correspond to each small section, and (**c**) corresponding activation of index shown in Figure 7a and thumb in Figure 7b are shown in shaded green in simulator matrices in the GUI.

In this study, we used Processing software to develop five 4 × 4 matrices for the tip of every digit of the hand, as shown in Figure 7. Each 4 × 4 matrix has 16 small sections corresponding to the 16 tactile pins of each P20 Braille cell. Figure 7a,b show the actual tactile pins activated for the index finger and thumb matrices corresponding to the green circles in the index and thumb matrices of the GUI shown in Figure 7c. Each matrix can hover to an image on the computer screen using the Leap Motion Controller. The tactile matrices in our simulator can be grouped and be moved using a computer mouse using the coordinates of its pointer as a reference. The tactile matrices can also be anchored on the fingertips and wrist coordinates extracted in real-time by the hand-tracking algorithm of the Leap Motion Controller. Each small section in every tactile matrix simulator is an ROI that computes the amount of black or white pixel. The simulator can be used to scan the surface or the edges of binary image using the Canny edge detector algorithm. If the small section of the tactile matrix simulator is more than 50% black, it will be filled with the color green, and an “on” or “up” signal is sent by the microcontroller to the assigned tactile pin. On the other hand, if the small section is less than 50% black, an “off” or “down” signal is sent by the microcontroller to pull down the assigned tactile pin. The flowchart of surface scanning and edge detection algorithm is shown in Figure 6.

## 4. Experimental Setup

**Figure 8:**
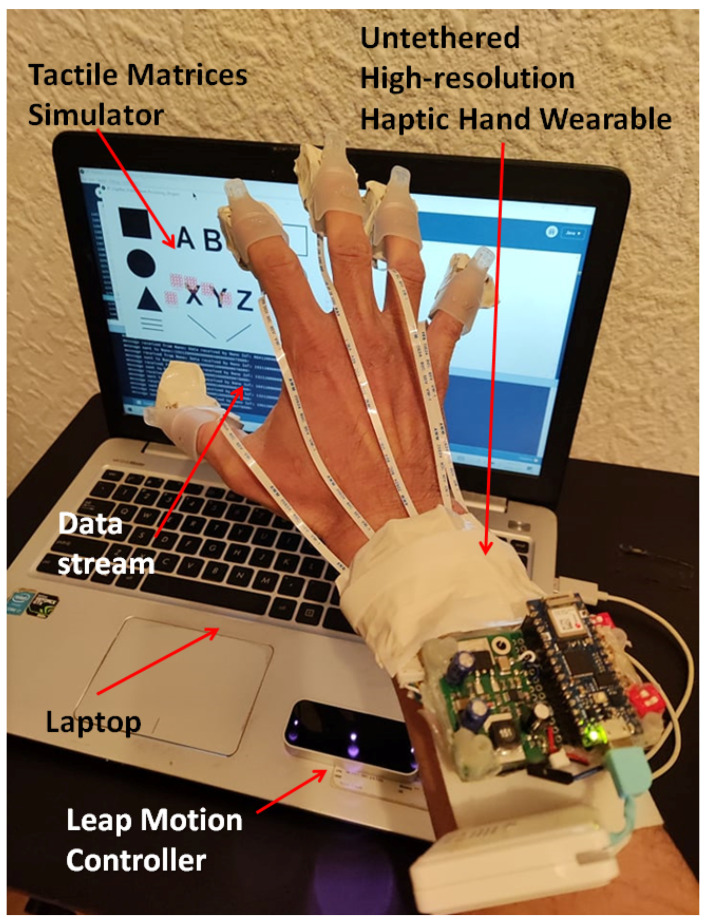
The integrated experimental setup for 2D scanning.

**Figure 9:**
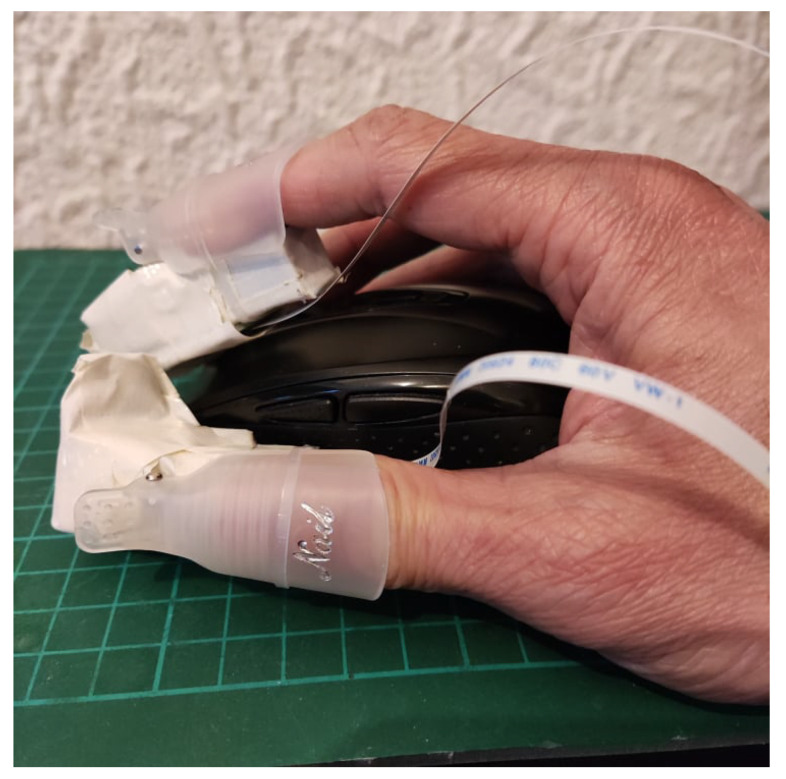
How a mouse can be a tactile mouse: when the user wears our untethered haptic hand wearable.

The integrated experimental setup is shown in Figure 8. The high-resolution haptic hand wearable is wirelessly connected via classic Bluetooth or WiFi. The hand will hover to a Leap Motion Controller that will track the hand pose and positions of the fingertips. The six 4 × 4 tactile matrices in the simulator are pegged on the coordinates of the fingertips and wrist. As the hand or fingers move, the tactile matrices in the simulator move across the computer display. The simulator tracks the changes in each of the tactile matrices and sends a corresponding signal to the microcontroller to activate or deactivate the corresponding tactile actuator in the haptic hand wearable. Aside from Leap Motion Controller, our fingertip tactile matrix simulator can be driven using a mouse, as shown in Figure 9. Our simulator and wearable haptic prototype have the novelty of easily converting any computer mouse into a tactile mouse similar to the tactile mouse by Watanabe et al. [63], Hribar et al. [64], and Owen et al. [65]. The idea is when the surgeon hovers over the object on the screen and feels it by actuating fingertips, similar to how a surgeon would feel more subtle information via fingertips in open surgeries [42,43].

### Wireless Connection

**Figure 10:**
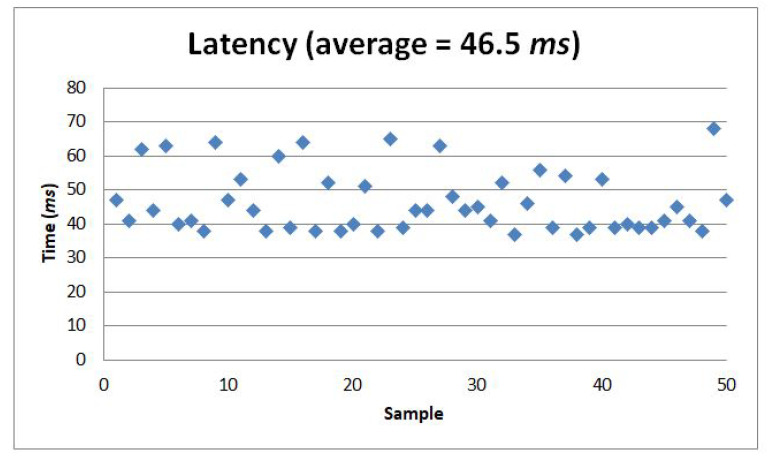
An average of 46.5 ms latency was measured from 50 samples.

The Arduino Nano 33 IoT uses a WiFi Nina chip to connect to WiFi, and there are many sample programs in the Arduino IDE to make the WiFi connection. Moreover, this microcontroller can connect via Bluetooth Low Energy (BLE) and classic Bluetooth. Unlike WiFi and BLE, which have many official and easy-step tutorials and sample codes for connection, the classic Bluetooth activation is not a straightforward process for the Arduino Nano 33 IoT. Some Arduino users shared their different procedures on how to activate the classic Bluetooth capability of Arduino Nano 33 IoT in the Arduino forum [66]. The classic Bluetooth is needed in this study instead of the BLE to make a wireless serial connection between the laptop and the haptic hand wearable. A Bluetooth connection does not need a hotspot or a server to link the haptic hand wearable to the laptop compared to a WiFi connection. We have successfully activated the classic Bluetooth running at a 115,200 baud rate. We measured latency by recording the sending time of the dataframe from our tactile matrix simulator to the hand wearable and the receiving time of acknowledgment message from the wearable’s microcontroller. We recorded 50 samples of data communications using our wearable. Using Bluetooth connection, we obtained an average latency of 46.5 ms with a standard deviation (SD) of 9.06 ms, as shown Figure 10. Our latency is a little higher than the 42.38 ms PC-Arduino USB serial communication latency reported in [67]. However, our setup is wireless with 46.5 ms latency which is much lower than the 600 ms tolerable delay acceptable by a surgeon in the absence of haptic feedback in teleoperated surgery reported by Tavakoli and Patel [10]. We have a larger SD of 9.06 ms as compared to the 1.29 ms SD in USB serial communication reported [67] because we have tested our latency using Bluetooth communication. An actual classic Bluetooth connection test video of our Arduino Nano 33 IoT can be found in this link [68].

## 5. Application Example

### 5.1. 2D Surface Scanning and Edge Detection

The prototype can scan a surface, as shown in Figure 7 and Figure 8 wherein all the tactors can be activated simultaneously when it covers a large area. Our previous work is limited to a single fingertip [48]. However, we expanded it into five fingers in this study. Actual scanning test videos can be found in this link [68].

**Figure 11:**
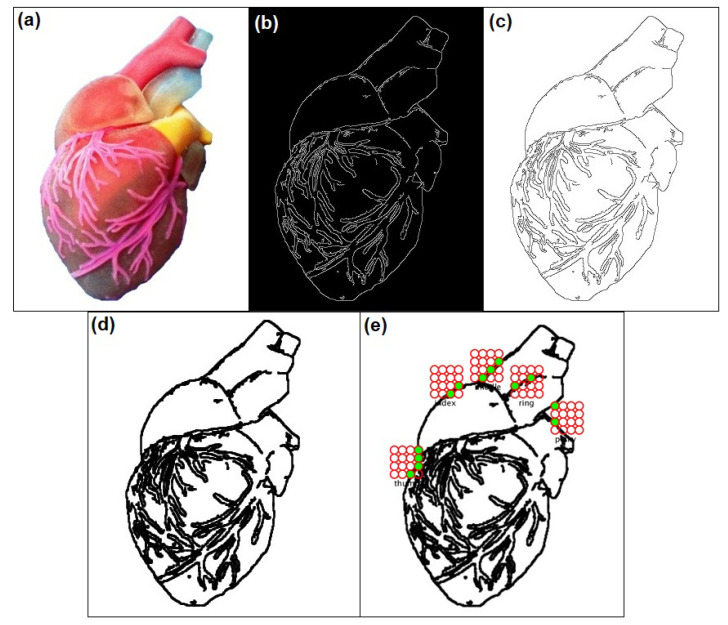
Edge detection using Canny edge algorithm with Hough transform to thicken the lines. (**a**) RGB image of a heart, (**b**) Canny edge result (white lines in black background), (**c**) inverted Canny edge, (**d**) thickened Canny edge using Hough transform, and (**e**) edge detection with tactile matrices simulator.

Aside from surface scanning, the prototype can also detect the edges of a given image. The edge detection process using Canny edge and Hough transform is shown in Figure 11. From the given RGB image of a heart, as shown in Figure 11a, the Canny edge detection algorithm is applied to produce the result as white edges in a black background, as shown in Figure 11b. There is a need to invert this Canny edge result because our prototype considers black color pixel as an “up” signal for the actuator. By simply using the image filter function “filter(INVERT)” without space, the Canny edge result is now in black lines with a white background as shown in Figure 11c. To increase the one-pixel thickness of the Canny edge image, Hough transform for lines can be used to achieve the image with thick lines shown in Figure 11d. The improved Canny edge image in white background is then fed as background in our tactile matrix simulator as shown in Figure 11e.

### 5.2. Tapping Vibration

**Figure 12:**
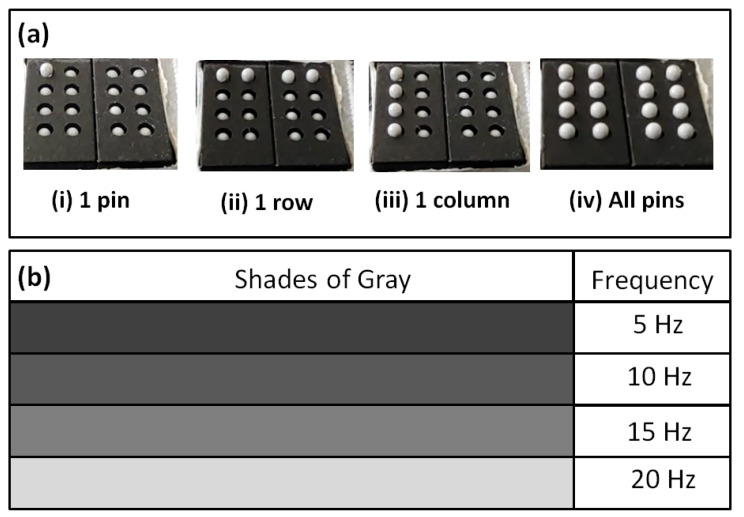
Tapping vibration. (**a**) Tapping vibration can be achieved using (**i**) 1 pin, (**ii**) 1 row, (**iii**) 1 column, and (**iv**) all pins. (**b**) Different tapping frequencies can be assigned to various shades of gray.

Another advantage of our prototype is that it can produce tapping or linear vibration on the fingertips. The tactile pins of P20 Braille cells can be activated in a tapping manner and controlled using a pulse signal at different frequencies and duration. We tried vibrating the tactile pins using 5 Hz, 10 Hz, 15 Hz, and 20 Hz frequencies, which are within the range of frequencies that triggers fingertip’s tactile mechanoreceptors: Meissner’s corpuscles and Merkel’s cells that are sensitive to edge pressure and flutter tap as reported by Visell [19]. Our prototype can produce point tapping by activating one pin, line tapping by activating a row, a column, and area tapping by activating all the pins in the 4 × 4 fingertip tactile matrix as shown in Figure 12a(i–iv), respectively.

In one tapping test we conducted, the topmost row vibrates at 5 Hz for 2 s, followed by succeeding rows going down with frequency increments of 5 Hz for each row. Rows or columns can execute the tapping pattern with increasing or decreasing tapping frequency. Aside from activating the pins by row or column, we can vibrate all 16 pins simultaneously. By doing this, we can have a larger tapping area compared to a column or row vibration.

Moreover, instead of directly programming the pins to vibrate at a specific frequency, we can use another parameter that will dynamically change the tapping frequency. Different tapping frequencies can be assigned to various shades of gray, as shown in Figure 12b. As the ROI in the tactile simulator moves across a grayscale image, the tactile actuator is activated with the corresponding tapping frequency. The actual tapping test videos can be found in this link [68].

**Figure 13:**
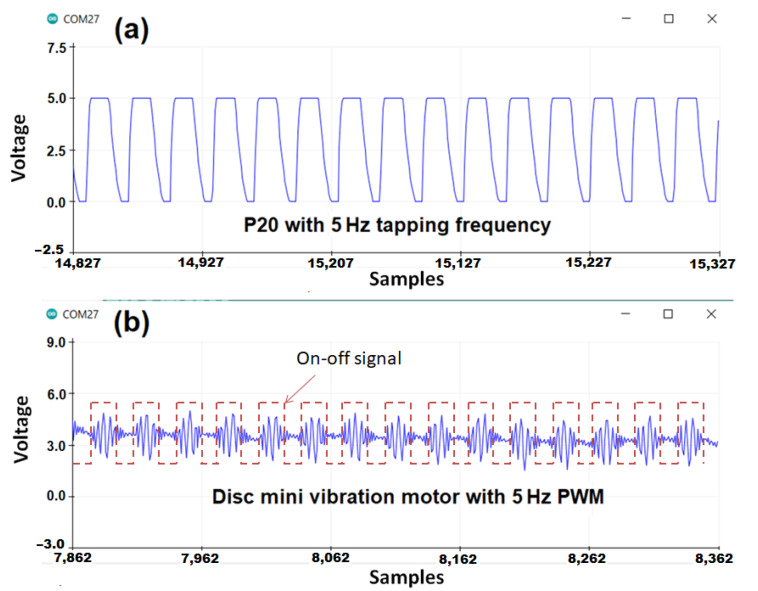
Tapping vs buzzing vibration. (**a**) Tapping: 5 Hz vibration has clear and distinct vibrations. (**b**) Buzzing: The low-frequency 5 Hz on–off signal for the vibration motor (in red dotted lines) has a high-frequency buzzing vibrations (in blue spikes). The inherent high-frequency vibrations within low-frequency on-time is due to the rotating mass inside the vibration motor running at 14,000 rpm or around 230 Hz.

The actuators at the fingertips of our prototype can produce clear and distinct tapping vibrations compared to ERM and LRA vibrotactiles with multiple frequencies combining low modulating frequency for the pattern and the inherent high-frequency due to their vibrating mass [69]. Each tactile pin in our 4 × 4 fingertip tactile matrix can vibrate up to 300 Hz, similar to the dynamic response of Metec P16 Braille cell reported by Owen et al. [65]. Comparison of tapping and buzzing vibrations are shown in Figure 13. ERM and LRA motors have intrinsic high-frequency vibrations based on their moving mass, but they can have another vibration based on how we turn them on or off. Our prototype can produce point, line, and area tapping vibrations that cannot be replicated using vibrotactile motors. When applied to haptics, the multiple vibrations on the ERM and LRA motors are more complex than the tapping vibrations that our prototype can produce. Our prototype could be used as a test platform for future studies related to tapping vibrations perception on the fingertips based on a grayscale image or contour map. We argue that this tapping frequency is another added feature to convey fine-grained information with more clarity to the surgeon/user, especially in laparoscopy.

### 5.3. 3D Surface Scanning and Edge Detection Using Oculus Quest2 VR Headset

**Figure 14:**
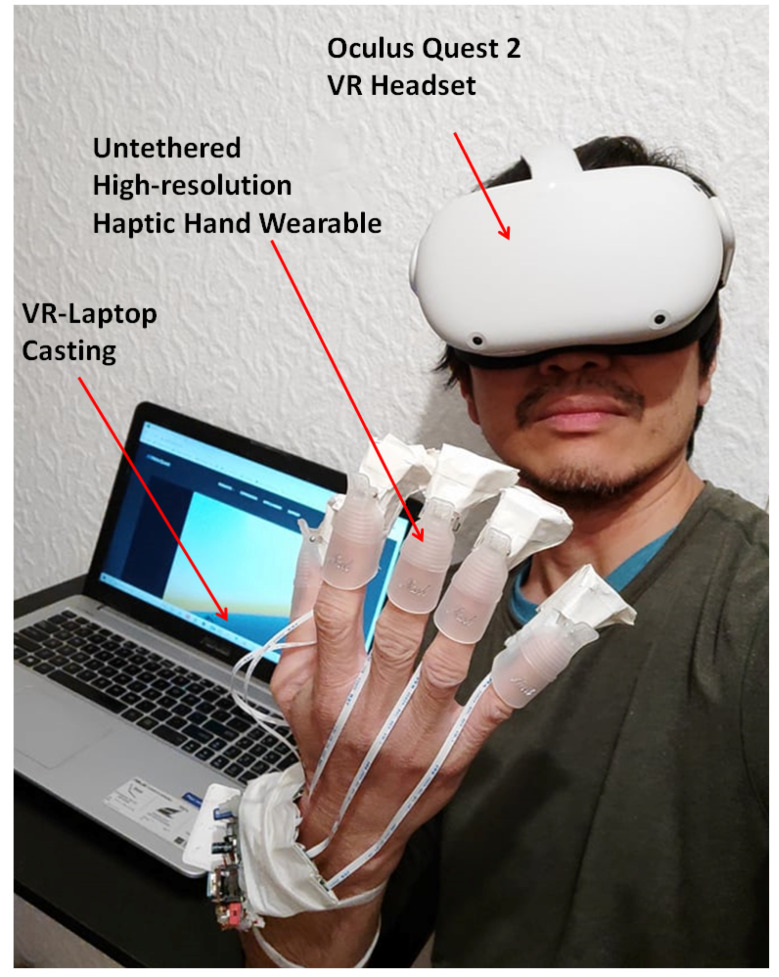
Setup for 3D surface scanning of VR objects using Oculus Quest 2 VR headset.

Our high-resolution haptic wearable can be connected to an Oculus Quest 2 VR headset using a Bluetooth connection, and the VR environment can be cast on a laptop, as shown in Figure 14. The 3D virtual objects are developed using Unity software. Unlike the open-palm setup in Figure 8 where the Leap Motion Controller hand tracking device is at the bottom, the position of the control circuit of the haptic wearable was placed on the wrist so that the Oculus Quest 2 VR headset can track the back of the hand.

**Figure 15:**
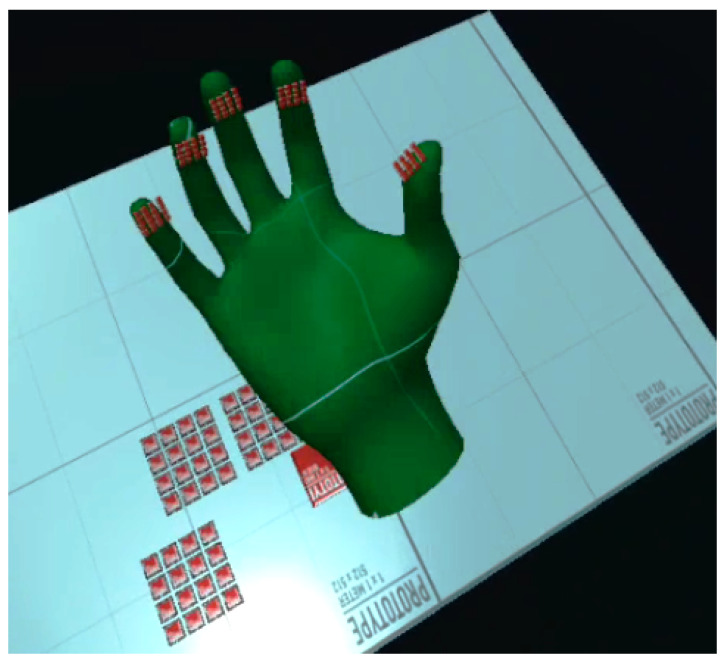
Scaling of the VR tactile actuators.

**Figure 16:**
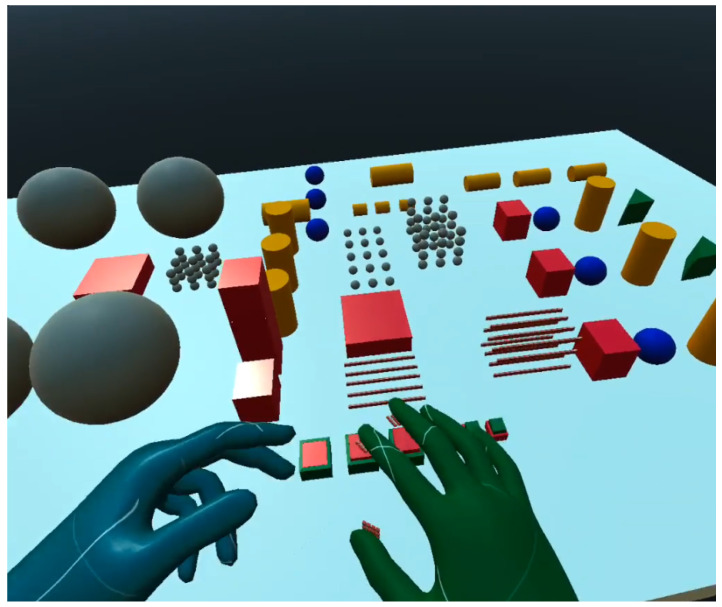
3D VR environment.

**Figure 17:**
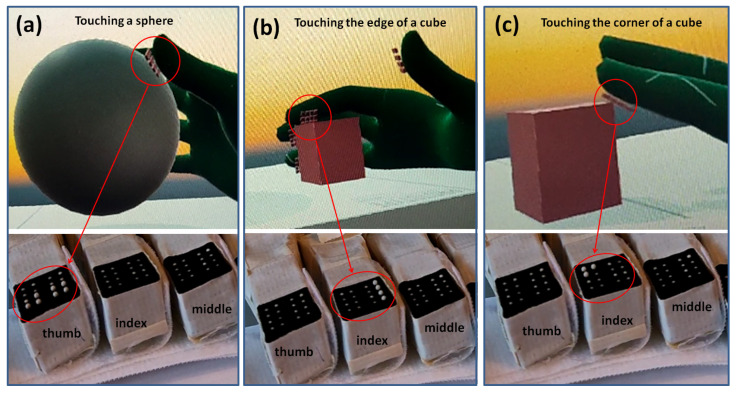
Touching 3D VR objects. (**a**) Touching the surface of a VR sphere, (**b**) touching the edge of a cube, and (**c**) touching the corner of a cube.

Similar to the 2D tactile matrix simulator, we created a 3D tactile matrices simulator, as shown in Figure 15. The basic idea is to create a group of 4 × 4 button switches and attach it to each of the fingertips of the VR hand avatar. A tutorial on how to create a simple VR button switch can be found here [70]. We created a 3D environment, as shown in Figure 16, where we can explore the shapes and edges of 3D VR objects. Each button switch on the fingertip of the 3D VR hand has a script that sends a signal to the corresponding hardware actuator during VR object collision, as demonstrated in Figure 17. The binary scale of either high or low pin actuation was presented and demonstrated to feel the contours of 3D VR objects as shown in Figure 16. The participants felt basic contours such as a sphere, edge, and corner of a cube in the 3D screen, as shown in Figure 17, when they wore the Oculus Quest 2 VR Headset. The actual 3D VR test videos can be found in this link [68].

### 5.4. Experiment Protocol

In all experiments, human participants signed written consent form approved by the ethics committee of Liverpool Hope University (LHU Approved Ethical Clearance: S–19-10-2021 SEL 5256).

## 6. Experiments Involving Human Participants

Although our prototype is not intended for vision substitution but for tactile augmentation to complement vision, three tactile pattern recognition experiments were carried out in this study involving nine human participants. We conducted three experiments involving human participants in this study. Experiment 1 is for a spatial test to understand a human’s ability to recognize the tactile patterns activated at different locations on the fingertip. Experiment 2 is for a temporal test to understand a human’s ability to recognize different vibrating patterns moving in different directions on the fingertip. Experiment 3 is for a 2D image recognition test to understand a human’s ability to recognize the lines, simple shapes, and edges.

### 6.1. Experiment Procedure

Nine right-handed subjects (seven males, two females with ages 23–60 years old) participated in the experiments after giving informed consent. The Dutch Handedness Questionnaire [71] was used to evaluate the handedness. Subjects were asked to use their most dominant hand to feel or perceive the tactile patterns on their index finger and or thumb. Patterns or activating signals were sent wirelessly from the laptop to our haptic wearable using Bluetooth communication, as shown in Figure 8. When the pins were actuated, subjects were then asked what pattern they felt based on the printed patterns given to them, which are similar to the patterns in the graphical user interface (GUI) for each test. The experimenter records the responses of the participants by clicking the respective pattern on the GUI. A log file is generated for each test. The log file contains played patterns and the subject’s selection on GUI after each trial.

#### 6.1.1. Experiment 1: Spatial Test

**Figure 18:**
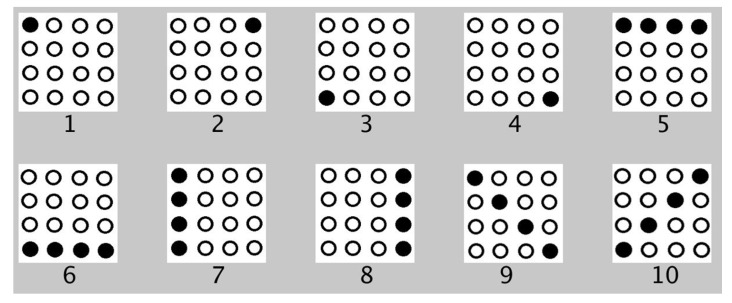
Patterns for spatial test.

Using the index finger, each human participant was trained before the test. Ten different patterns related to the perimeter, corners, or boundaries of the tactile matrix were activated, and were run in series or in the same sequence as shown in Figure 18. After the training, the ten patterns were played pseudo-randomly. The spatial test was further divided into two parts: (a) patterns were activated once, and (b) patterns were activated three times for each trial. There were three repetitions for each trial. Therefore, 30 trials were tested for single activation and 30 trials for 3 × activation. The participants were then asked what patterns they perceived on their index finger based on the patterns shown in Figure 18 for each trial.

#### 6.1.2. Experiment 2: Temporal Test

**Figure 19:**
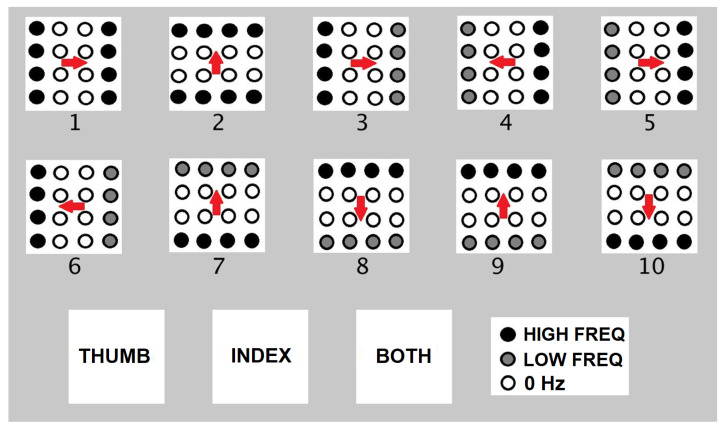
Patterns for temporal test.

In contrast to the spatial test, wherein the activated pattern is in a fixed location within the matrix for each trial, patterns with different frequencies move in different directions across the matrix in the temporal test. This temporal test is further divided into two parts: (a) using only the index finger, and (b) using the index finger and/or thumb. Each human participant is trained before the test by running in sequence ten different patterns having different frequencies (20 Hz and 10 Hz) and different directions of movements, as shown in Figure 19. Each pattern can be activated for the index finger only, for the thumb only, and for both the index finger and thumb simultaneously. During the testing, patterns were played pseudo-randomly, and there were three repetitions for each trial. The participants were then asked what patterns they perceived on their index finger based on the patterns shown in Figure 19 for each trial.

#### 6.1.3. Experiment 3: 2D Scanning Test

**Figure 20:**
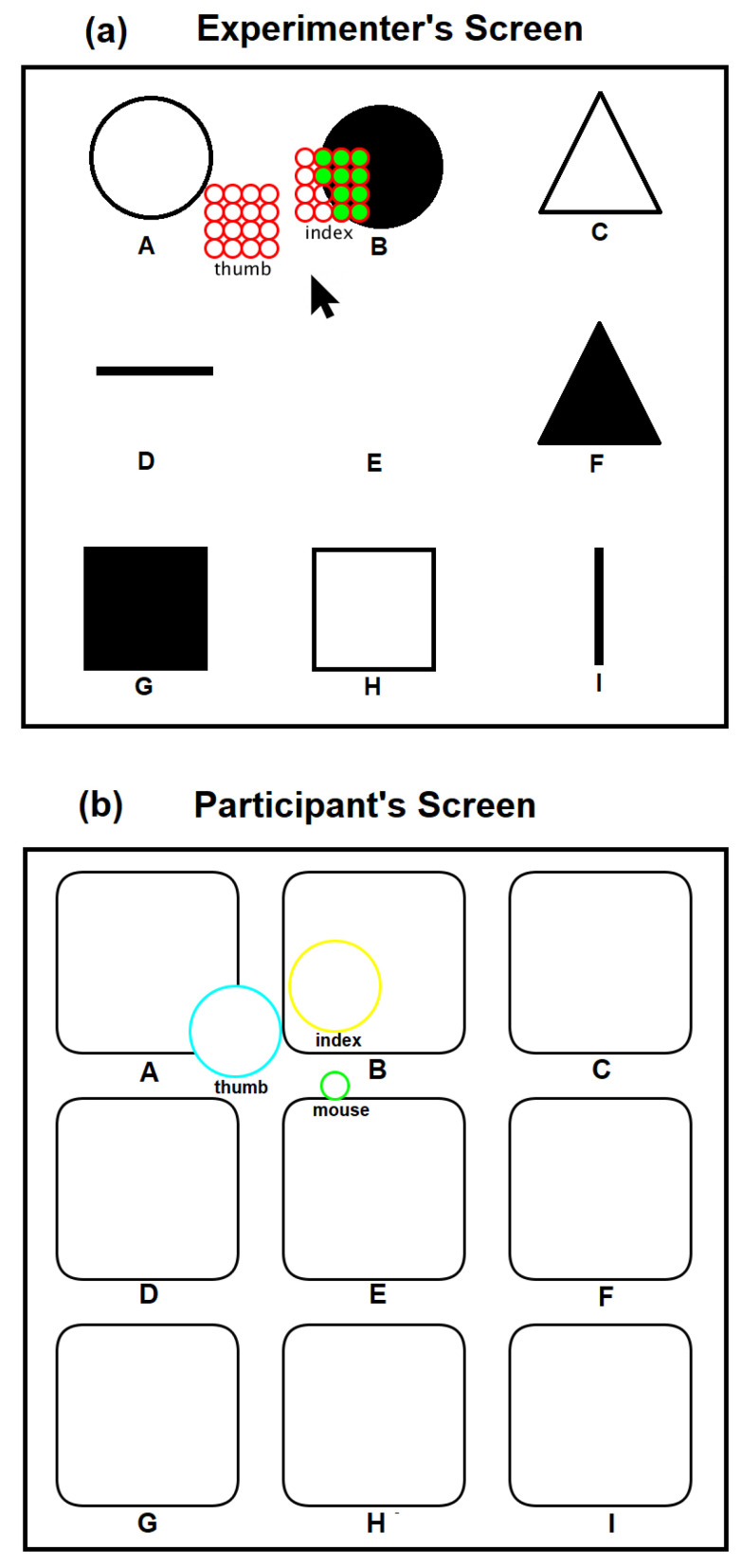
Patterns for 2D scanning test. (**a**) Experimenter’s screen, (**b**) participant’s screen. Participants were able to see empty boxes to explore and identify the objects A to I.

**Figure 21:**
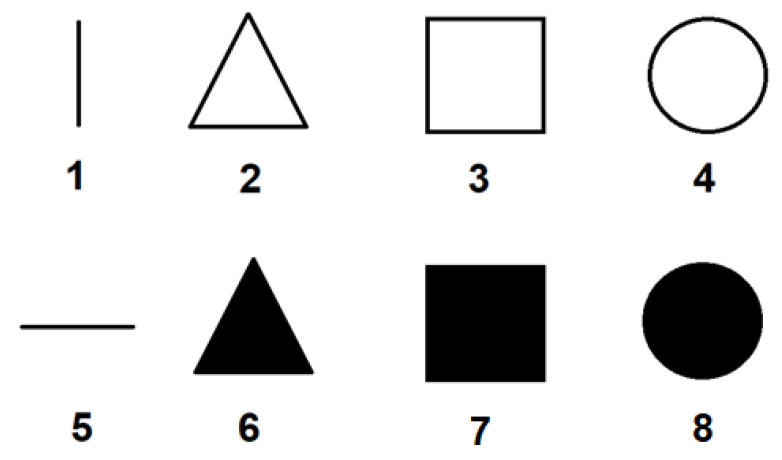
2D training patterns.

In the 2D scanning test, a setup similar to Figure 8 is used but each participant used a computer mouse, as shown in Figure 9, to move the fingertip tactile matrices in the simulator, as shown in Figure 20. This experiment used two computer screens: one for the experimenter, and one for the participant. During the training, the patterns using horizontal and vertical lines, and geometric figures, such as triangle, square, and circle, in edge or plane figures as shown in Figure 21 are displayed on the computer screen where each participant can see. Each participant is then asked to feel the tactile pin actuation on the index finger and thumb as the mouse is moved across the edges or surface of the patterns in the computer display. During the testing, the patterns in Figure 21 are placed in different order, as shown in Figure 20, which are visible to the experimenter but are concealed to the participants. The participants can only see the nine boxes in the participant’s screen, as shown in Figure 20, with blue and yellow circles corresponding to the thumb and index tactile matrices, respectively. Moreover, the small green circle corresponds to the mouse pointer which guides the participant during the scanning. Each participant is then asked to move their hand on top of the mouse to scan or explore each of the nine boxes and tell the experimenter what geometric figure or line they can perceive. The box can have a line or geometric shape, but it can also be empty or blank.

### 6.2. Experiment Results and Discussion

#### 6.2.1. Experiment 1: Spatial Training

**Figure 22:**
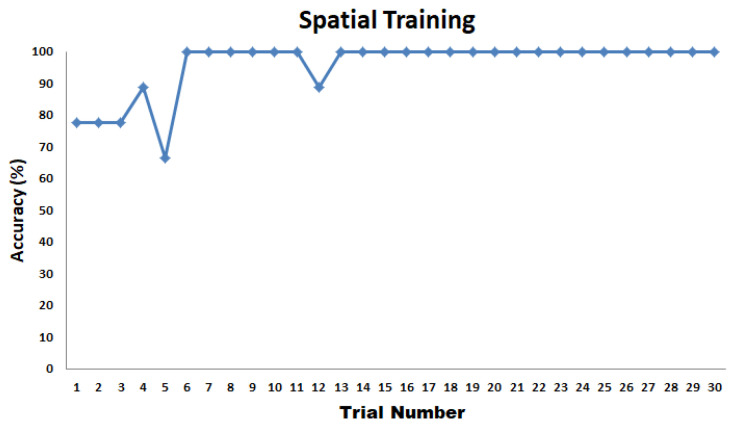
Spatial training results.

On average, the participants were able to recognize the different patterns shown in Figure 18 with 95.93% accuracy. The average test time is 2 min and 2 s with an SD of 37 s. Participants were trained for 30 trials. There was an improvement in the accuracy from 77.78 to 100% with respect to the 30 trials, as shown in Figure 22. After 12 trials, participants’ accuracy of recognition reached 100%.

#### 6.2.2. Experiment 1: Spatial Test

**Figure 23:**
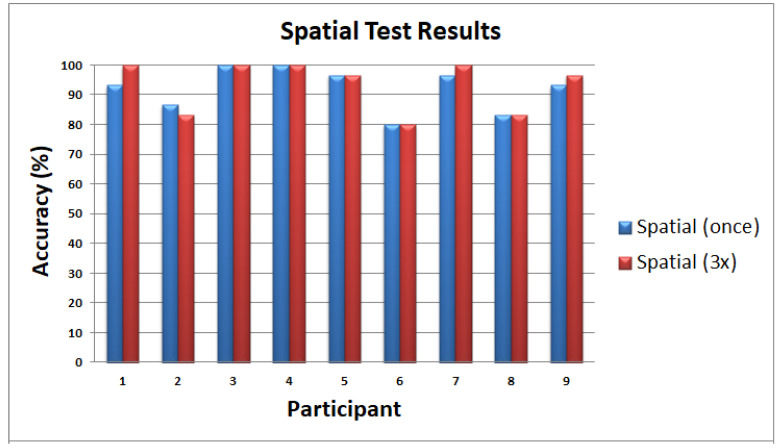
Spatial Test Results.

The spatial test results for part 1 and part 2 graphed side by side are shown in Figure 23. The average test time to complete the first part of Experiment 1 is 2 min and 25 s with an SD of 35 s. The test time for the second part of Experiment 1 is 2 min and 43 s with an SD of 10 s. The results show that the perception of patterns activated thrice has very minimal improvement compared to the situation when the patterns are activated once. On average, participants were able to recognize different patterns related to corners, diagonal, perimeter, or boundaries of the tactile matrix with 92.22% accuracy for patterns activated once, and 93.33% accuracy for patterns activated three times. In general, it was noticed that, on average, tapping three times or a single time does not affect the responses. This would be useful in fast responses of humans in minimally invasive surgeries to react in real time without time delay. Moreover, the results show that single tapping is sufficient to feel the patterns that we played.

#### 6.2.3. Experiment 2: Temporal Training

**Figure 24:**
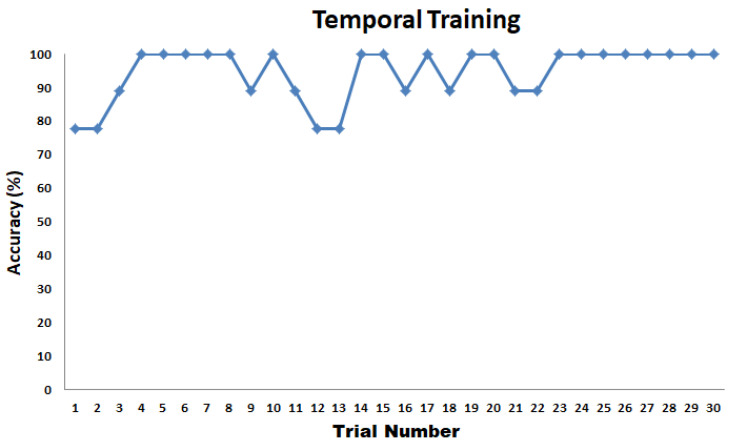
Temporal training results.

On average, the participants were able to recognize the different patterns shown in Figure 19 with 94.44% accuracy. The average test time is 9 min and 15 s with an SD of 1 min and 56 s. Participants were trained for 30 trials. There was an improvement in the accuracy from 77.78 to 100% with respect to the 30 trials, as shown in Figure 24. After 22 trials, participants’ accuracy of recognition reached 100%.

#### 6.2.4. Experiment 2: Temporal Test

**Figure 25:**
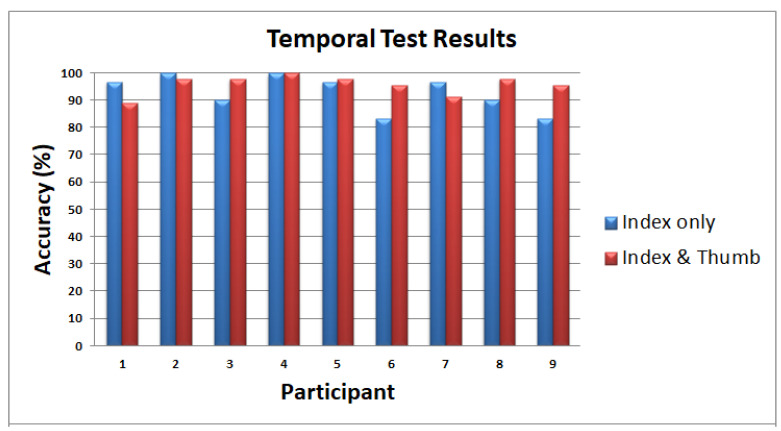
Temporal test results (index only).

The results of the temporal test for index finger only and the temporal test for index finger and thumb are shown side by side in Figure 25. The average test time to complete the first part of Experiment 2 is 9 min and 18 s with an SD of 1 min and 41 s. The test time for the second part of Experiment 2 is 15 min and 6 s with an SD of 1 min and 3 s. In index-finger-only test, on the average, participants were able to recognize patterns vibrating at different frequencies moving in different directions with 92.96% accuracy. Moreover, the participants can clearly distinguish if the pattern is activated on the thumb only, index finger only, or on both index finger and thumb. On average, participants were able to recognize patterns with 95.80%. A single finger and double fingers exploration in temporal testing shows that a single finger is enough to recognize smooth surfaces/shapes. The combination of index and thumb helped to increase the accuracy from 92.96% to 95.80%.

#### 6.2.5. Experiment 3: 2D Scanning Training

In the 2D scanning training, the participants are free to explore the different 2D geometric figures shown on the computer screen. On average, the exploration time for the whole training is 2 min and 1 s with an SD of 22 s. Only the training time was recorded because the participants could directly see the 2D objects in the display during the training. The 2D patterns recognition accuracy is performed in the 2D scanning test.

#### 6.2.6. Experiment 3: 2D Scanning Test

**Figure 26:**
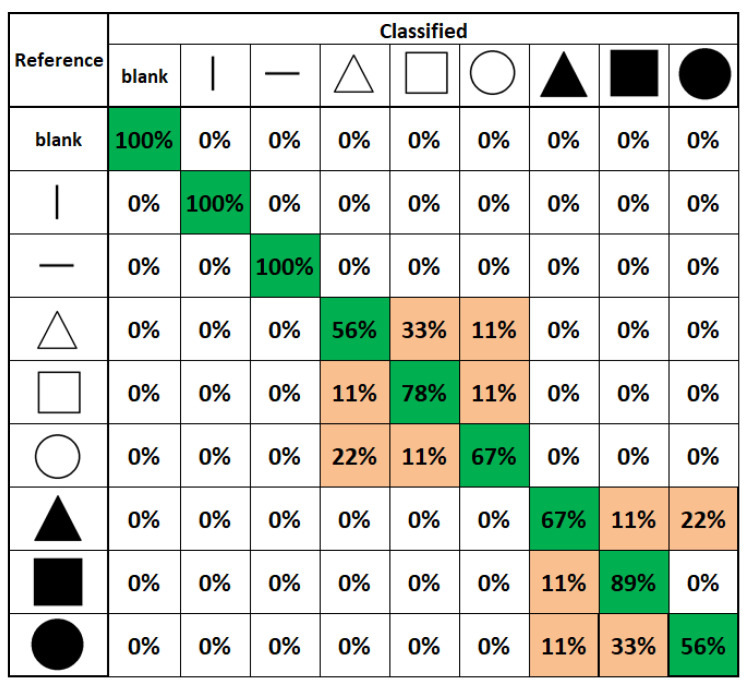
2D scanning test confusion matrix.

Using the index finger and thumb, all the human participants in the 2D scanning test were able to distinguish an empty/blank box, a vertical line, and a horizontal line, as shown in the confusion matrix of Figure 26 with 100% accuracy. Moreover, participants were able to achieve a higher detection rate of 89% and 78% in detecting plane and edge square, respectively. The lowest accuracy is 56% for edge triangles, confused mostly with edge squares. We also obtained a 56% average accuracy in recognizing plane circles that confuses mostly with the plane square, not with vertical or horizontal lines or blank squares. However, the authors argue that given the higher accuracy percentage values, humans need to be trained for our novel wearable to achieve techniques in recognition, as naive participants’ recognition values are promising. Since surgeons are trained for surgeries, this system would be easily adapted to enhance techniques in laparoscopic surgeries. Furthermore, these 2D scanning test results show that our prototype can be used as a vision substitution device as well. On average, the exploration time to complete the 2D scanning test is 4 min and 11 s with an SD of 32 s. The exploration time during testing is twice the training time.

Furthermore, 3D virtual experience from participants could be summarized as follows: all the participants reported that they felt the shapes, contours, or edges of a 3D object shown in Figure 16. They also reported that they felt the contours or edges at the back of a 3D VR object even if it was not visible on the screen. This feeling of touching the back of a 3D VR object that is not visible on a 2D screen creates the illusion of touching a real 3D object in the VR world.

## 7. Limitations of the Current Design and Possible Future Improvements

Though our current prototype design is focused only on high-resolution cutaneous feedback, we can still improve it in the near future to make it a multi-modal haptic feedback hand wearable to integrate cutaneous feedback and kinesthetic feedback. Without compromising the fine-grained untethered portability design of our current prototype, we can add force feedback made from a low-cost ID badge holder and temperature feedback using a small thermoelectric generator (TEG) that we demonstrated in our previous studies related to multi-modal haptic hand wearable [9]. Our current design is limited to a single intensity in actuation. We are planning to explore different pin height activation to introduce different intensities by applying the method demonstrated by Headley and Pawluk [72] in 2010, wherein they vary the tactile pin height of a P16 Metec Braille cell by varying the supply voltage. The proposed above improvement would bring us to have an untethered multi-modal portable haptic hand wearable with high-resolution cutaneous feedback, force feedback, temperature feedback, and varying tactile pin height activation capability all in one device.

## 8. Conclusions and Recommendation

This study presents the design and development of a novel high-resolution haptic hand wearable with 80 tactile actuators. The characteristics of our prototype are wearable, portable, modular, and can be wirelessly connected using classic Bluetooth or WiFi. The components for the prototype are commercially available and the software application was developed using open-source software. Our wearable haptic device has a maximum power consumption of 830 mW and has a total weight of 204 g. Our prototype can be used to scan a binary image or detect edges of an image by using the Canny edge detection algorithm. Moreover, it can be fed with predetermined patterns such as the Braille patterns or tapping patterns. This would be useful to enhance multi-modal perception in minimally invasive surgeries. Since the current trend is more focused on kinesthetic feedback studies, our wearable would add more subtle information to the surgeon, similar to open surgeries.

Moreover, experiments involving human participants were conducted. In the spatial test, participants were able to recognize different patterns related to corners, diagonal, perimeter, or boundaries of the tactile matrix with 92.22% accuracy for patterns activated once, and 93.33% accuracy for patterns activated three times. In general, it has been noticed that on average, tapping three times or a single time does not affect the responses. In the temporal test, where index finger only was used, participants were able to recognize patterns vibrating at different frequencies moving in different directions with 92.96% accuracy, but the combination of index and thumb helped to increase the accuracy from 92.96% to 95.80%. In the 2D scanning test, participants achieved 100% accuracy in recognizing empty/blank box, vertical line, and horizontal line. Participants in the 2D scanning test were able to achieve detection rate of 89% and 78% in detecting plane and edge square, respectively. The lowest accuracy is 56% for edge triangles that were confused mostly with edge squares. We also obtained a 56% average accuracy in recognizing plane circles that confused mostly with the plane square, not with vertical or horizontal lines or blank squares. These 2D scanning test results show that our prototype can be used as a vision substitution device as well.

Furthermore, our prototype was developed together with Oculus Quest 2 VR headset to scan and feel the contours of a 3D VR object developed using Unity software. Participants felt basic contours such as a sphere, edge, and corner of the cube in the 3D screen when they wore the Oculus Quest 2 VR headset. We will explore more shapes and different environment testing in 3D VR in the future using our wearable. Therefore, our studies would shed light on other commercially available haptic gloves in the market to enhance cutaneous feedback. We believe that prototypes such as this are increasingly becoming a vital component part, and indeed a major contributing factor, of the success or otherwise of the emergent field of spatial computing.

## Figures and Tables

**Table 1:** Comparison of our “4 × 4 Fingertip Tactile Matrix” and “Untethered High-Resolution Haptic Hand Wearable”.

	Initial 4 × 4 Fingertip Tactile Matrix [48]	Current Untethered High-Resolution Haptic Hand Wearable
Type of tactile actuator (tactor)	Dot Braille cell	P20 Braille cells
Technology used in the tactor	Electromagnet (solenoid)	Piezoelectric bimorph bender actuator
Number of tactors	16 electromagnet-based pins	80 piezo-based pins
Type of wearable	1 fingertip	5-finger exoskeleton haptic glove
Mode of data transfer	USB wire	USB wire or wireless (Bluetooth or WiFi)
Microcontroller	Arduino Mega 2560	Arduino Nano 33 IoT
Tactor driver	MX1508 (H-bridge motor driver)	HV509 Shift Register for P20 Braille cells
Supply Voltage	5 V DC	5 V DC (DC–DC boost to 200 V for P20 Braille cells)
Application software	Python	Processing, Unity
Tactile simulator controller	Mouse	Mouse, Leap Motion Controller, Oculus Quest2

## Data Availability

High Resolution Haptic Glove Database. Available online: https://drive.google.com/drive/u/1/folders/1jBfT5-lH9-dx2a5O3dPrMOJ_ZvB8WX5z (accessed on 6 February 2022).
